# Dietary Selenium-Enriched *Bacillus subtilis* Improves Feed Utilization and Modulates Selected Responses to LPS Challenge in Laying Hens

**DOI:** 10.3390/ijms27198778

**Published:** 2026-09-30

**Authors:** Qiaoqiao Han, Yaru Wang, Yiyang Chen, Huiying Li, Yue Wang, Xiaotong Li, Qiugang Ma, Haihua Zhang, Shimeng Huang, Hongli Liu

**Affiliations:** 1 Hebei Key Laboratory of Specialty Animal Germplasm Resources Exploration and Innovation, College of Animal Science and Technology, Hebei Normal University of Science and Technology, Qinhuangdao 066004, China; hanqq12306@163.com (Q.H.); zhh83@126.com (H.Z.); 2 State Key Laboratory of Animal Nutrition and Feeding, College of Animal Science and Technology, China Agricultural University, Beijing 100193, China; wyrsmile0930@163.com (Y.W.); yiyang04020@163.com (Y.C.); 13898367985@163.com (H.L.); wangy07224@163.com (Y.W.); lixiaotong1117@163.com (X.L.); maqiugang@cau.edu.cn (Q.M.); 3 Laboratory of Feed Grain Safety and Healthy Poultry Farming, Beijing Jingwa Agricultural Science and Technology Innovation Center, Beijing 101206, China; 4 College of Animal Science and Veterinary Medicine, Jinzhou Medical University, Jinzhou 121001, China

**Keywords:** selenium-enriched *Bacillus subtilis*, laying hens, feed utilization, LPS challenge, barrier-related gene expression, cecal microbiota

## Abstract

This study evaluated the effects of dietary selenium-enriched *Bacillus subtilis* (HBS) supplementation on laying performance, egg quality, and responses to lipopolysaccharide (LPS) challenge. A total of 120 Jinghong-1 laying hens received a basal or HBS-supplemented diet for 12 weeks. Subsequently, hens within each dietary replicate were allocated to phosphate-buffered saline (PBS)- or LPS-injected subgroups, and one hen per subgroup was sampled 12 h after injection, yielding six sampled hens per treatment combination. HBS supplementation reduced the feed-to-egg ratio (1.96 vs. 2.15, *p* = 0.023) and increased albumen height (7.17 vs. 6.60 mm, *p* = 0.003) and Haugh unit (83.01 vs. 78.65, *p* = 0.001). Compared with LPS, HBS+LPS showed lower serum IL-1β concentrations, higher CAT and T-SOD activities, greater jejunal villus height and villus height-to-crypt depth ratio, and higher expression of selected antioxidant- and tight-junction-related genes. Jejunal cytokine responses varied among genes. No significant differences in the measured cecal alpha-diversity indices were detected, whereas exploratory LEfSe analysis identified several treatment-associated taxa. Cecal butyrate concentration was higher in HBS than in CON, with no significant difference between HBS+LPS and LPS. Collectively, these findings indicate that HBS supplementation improved feed utilization and albumen quality and was associated with changes in selected antioxidant, inflammatory, and intestinal responses following LPS challenge. The contributions of selenium and *B. subtilis* could not be distinguished.

## 1. Introduction

Intestinal health is central to nutrient utilization, egg production, and resilience to inflammatory stress in laying hens. Nutritional strategies that support intestinal homeostasis may help maintain production efficiency under challenging conditions.

The gastrointestinal tract is a dynamic interface connecting dietary nutrients, intestinal microorganisms, microbial metabolites, epithelial barrier function, and mucosal immunity. Maintenance of intestinal homeostasis requires coordinated regulation of microbial activity, epithelial renewal, immune responses, and microbial metabolites, particularly short-chain fatty acids (SCFAs) [1,2,3,4,5,6]. Disruption of this balance can result in oxidative stress, excessive inflammatory activation, epithelial barrier impairment, and reduced digestive and absorptive capacity. Lipopolysaccharide (LPS), a major structural component of the outer membrane of Gram-negative bacteria, is commonly used to establish experimental models of systemic and intestinal inflammatory stress in poultry. LPS exposure activates innate immune signaling, promotes the production of proinflammatory cytokines, disrupts redox homeostasis, and damages intestinal morphology and epithelial barrier integrity [7,8,9,10]. Therefore, the LPS-challenge model provides a useful approach for evaluating nutritional interventions designed to improve the resilience of laying hens to inflammatory stress.

Selenium (Se) is an essential trace element involved in antioxidant defense, immune regulation, thyroid hormone metabolism, and intestinal homeostasis. Many of its biological functions are mediated by selenoproteins, including glutathione peroxidases, thioredoxin reductases, and other redox-regulating enzymes [11,12,13,14]. Adequate Se intake supports cellular redox balance and immune competence, whereas inappropriate Se status may compromise antioxidant defenses or cause toxicity. Importantly, the biological efficacy, tissue retention, and safety of supplemental Se depend on its chemical form, dose, and bioavailability [12,13,14,15,16,17]. Compared with conventional inorganic Se sources, organically bound or microbially enriched Se may exhibit greater bioavailability and tissue retention under appropriate supplementation conditions [13,14,15,16,17]. Nevertheless, Se supplementation alone may not fully address the complex interactions among oxidative stress, intestinal barrier dysfunction, and microbial metabolism.

*Bacillus subtilis* is a spore-forming bacterium widely investigated as a probiotic feed additive because selected strains can survive feed processing and gastrointestinal transit, produce extracellular enzymes and antimicrobial compounds, and modulate intestinal immune and antioxidant responses [18,19,20,21,22,23,24,25]. Selenium-enriched *B. subtilis* is produced by cultivating the bacterium in the presence of Se, thereby combining a Se-containing microbial preparation with the biological properties of *B. subtilis*. In principle, the two components may provide complementary antioxidant, microbial, and host-directed functions; however, whether their effects are additive or synergistic can be established only by including Se-only and non-Se-enriched *B. subtilis* controls. The present experiment evaluated the combined preparation as a single feed additive and was not designed to resolve component-specific or synergistic effects. Studies in poultry have reported improvements in growth, antioxidant capacity, immune indices, intestinal morphology, and microbial composition after supplementation with Se-enriched probiotics or Se-enriched *B. subtilis* [26,27,28,29]. Recent probiotic studies and reviews further emphasize that effects on performance, intestinal morphology, barrier-related pathways, and microbial ecology depend on strain, dose, diet, and production context [30,31,32]. Evidence for the combined preparation in laying hens under inflammatory challenge remains limited.

Therefore, this study evaluated the effects of dietary HBS supplementation on laying performance and egg quality during a 12-week feeding trial, followed by assessments of organ indices, serum inflammatory and antioxidant parameters, intestinal morphology, jejunal gene expression, cecal microbiota, and short-chain fatty acid concentrations after PBS or LPS injection. We hypothesized that HBS would improve feed utilization and selected egg-quality traits and modify physiological responses to LPS challenge. Because selenium-only and non-selenium-enriched *B. subtilis* groups were not included, the experiment evaluated the HBS preparation as a whole.

## 2. Results

### 2.1. Effects of Dietary Selenium-Enriched Bacillus subtilis Supplementation on Laying Performance

Dietary HBS supplementation decreased the feed-to-egg ratio from 2.15 to 1.96 (*p* = 0.023), representing an approximately 8.8% reduction relative to CON (Table 1). Laying rate, average daily feed intake, average egg weight, and egg mass did not differ significantly between dietary treatments (*p* > 0.05).

### 2.2. Effects of Dietary Selenium-Enriched Bacillus subtilis Supplementation on Egg Quality

HBS supplementation increased albumen height from 6.60 to 7.17 mm (*p* = 0.003) and Haugh unit from 78.65 to 83.01 (*p* = 0.001; Table 2). No significant differences were detected in the remaining egg-quality parameters (*p* > 0.05).

### 2.3. Effects of Dietary Selenium-Enriched Bacillus subtilis Supplementation on Organ Indices

Liver index differed among the four groups (*p* = 0.025; Table 3). The value was lower in HBS+LPS than in LPS, although neither challenged group differed from CON. The gizzard index also differed among groups (*p* < 0.001); LPS had a higher value than CON, HBS, and HBS+LPS, whereas HBS+LPS did not differ from CON. Spleen, proventriculus, and kidney indices did not differ significantly among groups (*p* > 0.05).

### 2.4. Serum Inflammatory Cytokines and Antioxidant Enzyme Activities

**Figure 1:**
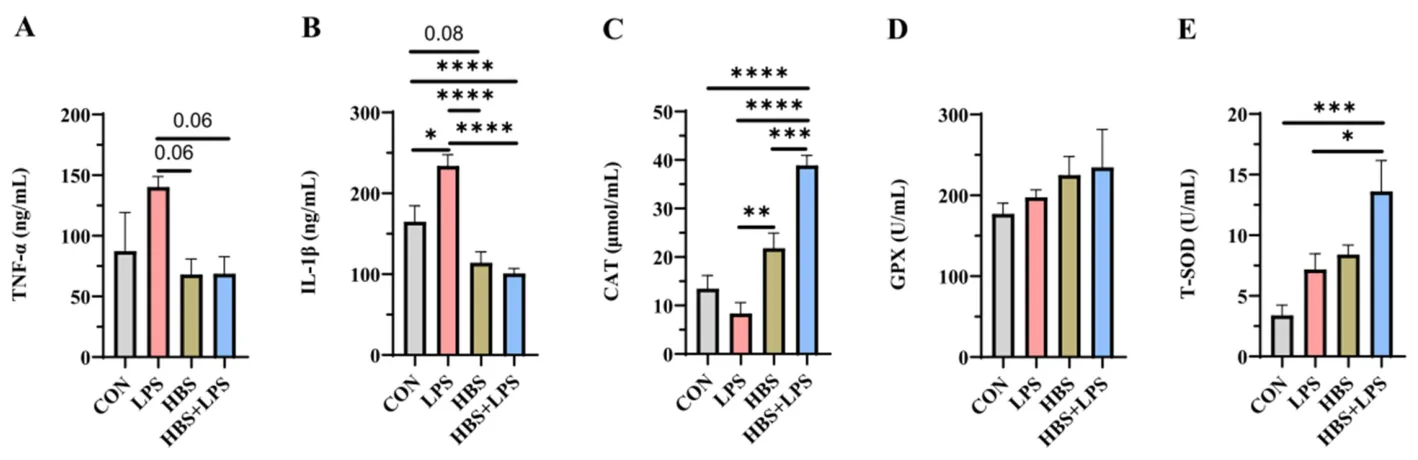
Effects of dietary supplementation with selenium-enriched *Bacillus subtilis* on serum inflammatory cytokines and antioxidant enzyme activities in LPS-challenged laying hens: (**A**) serum tumor necrosis factor-α (TNF-α) concentration; (**B**) serum interleukin-1β (IL-1β) concentration; (**C**) serum catalase (CAT) activity; (**D**) serum glutathione peroxidase (GSH-Px) activity; (**E**) serum total superoxide dismutase (T-SOD) activity. Data are presented as mean ± SD (*n* = 6). * *p* < 0.05, ** *p* < 0.01, *** *p* < 0.001, and **** *p* < 0.0001. CON, control; HBS, HBS supplementation; LPS, lipopolysaccharide challenge; HBS+LPS, HBS supplementation plus LPS challenge.

Serum CAT activity was higher in HBS than in CON (*p* < 0.01), whereas no significant differences were detected in TNF-α or IL-1β concentrations or in GSH-Px or T-SOD activities (Figure 1). Compared with CON, LPS increased serum IL-1β concentration (*p* < 0.05), whereas the difference in TNF-α did not reach statistical significance (*p* = 0.06). Compared with LPS, HBS+LPS showed lower IL-1β concentration (*p* < 0.0001) and higher CAT (*p* < 0.0001) and T-SOD activities (*p* < 0.05). No significant differences in GSH-Px activity were detected among the groups.

### 2.5. Intestinal Morphology

**Figure 2:**
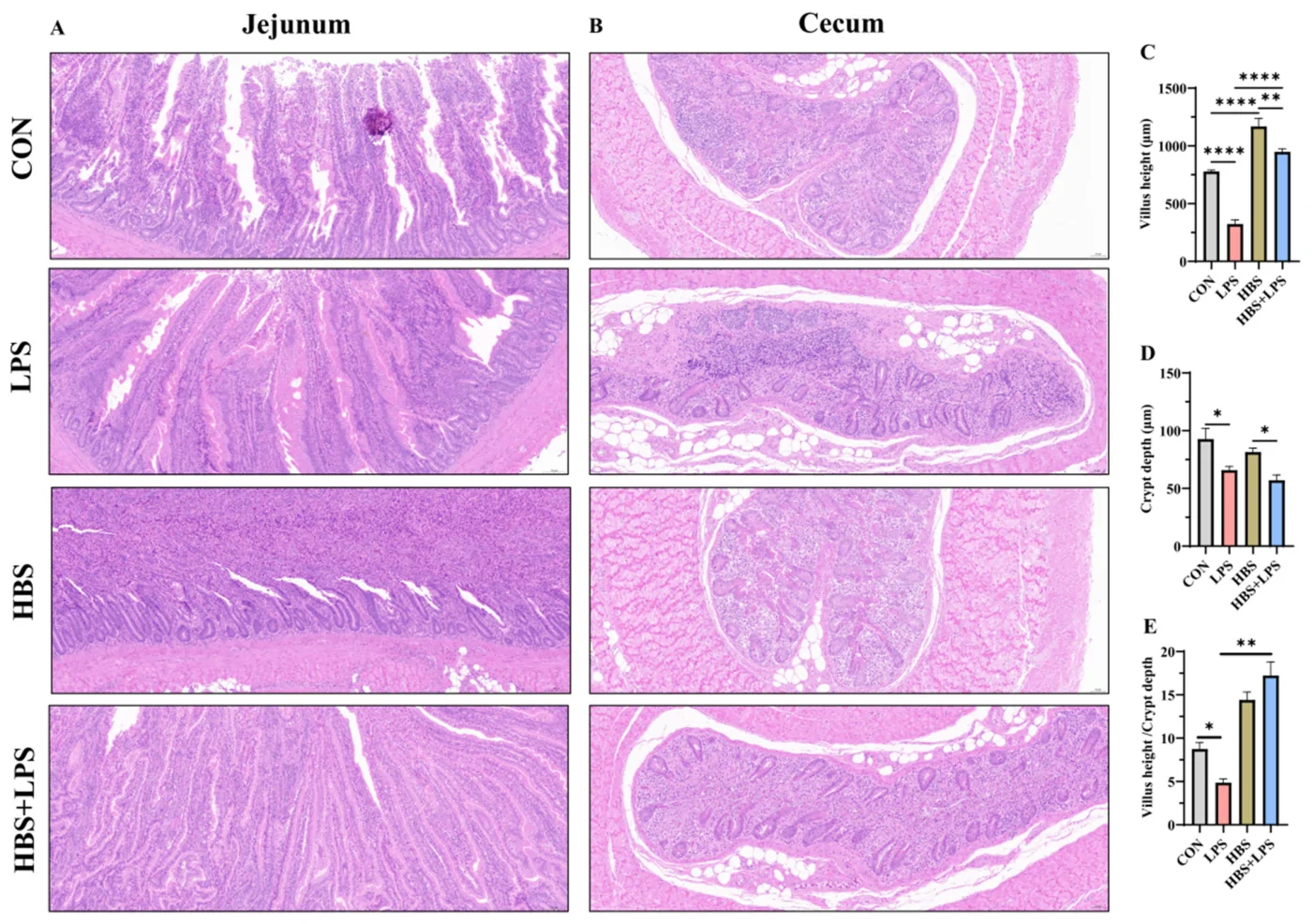
Effects of dietary supplementation with selenium-enriched *Bacillus subtilis* on intestinal morphology in LPS-challenged laying hens: (**A**) representative H&E-stained histological sections of the jejunum; (**B**) representative H&E-stained histological sections of the cecum; (**C**) villus height; (**D**) crypt depth; (**E**) villus height-to-crypt depth ratio (VH/CD). Data are presented as mean ± SD (*n* = 6). * *p* < 0.05, ** *p* < 0.01, and **** *p* < 0.0001. CON, control; HBS, HBS supplementation; LPS, lipopolysaccharide challenge; HBS+LPS, HBS supplementation plus LPS challenge.

Representative jejunal and cecal histological sections are shown in Figure 2. Jejunal morphometry revealed greater villus height in HBS than in CON (*p* < 0.0001). Compared with CON, LPS reduced villus height (*p* < 0.0001), crypt depth (*p* < 0.05), and the villus height-to-crypt depth ratio (*p* < 0.05). Compared with LPS, HBS+LPS showed greater villus height (*p* < 0.0001) and a higher villus height-to-crypt depth ratio (*p* < 0.01), without a significant difference in crypt depth. Representative cecal sections suggested less pronounced mucosal alterations in HBS+LPS than in LPS; this assessment was qualitative.

### 2.6. Cecal Microbiota

**Figure 3:**
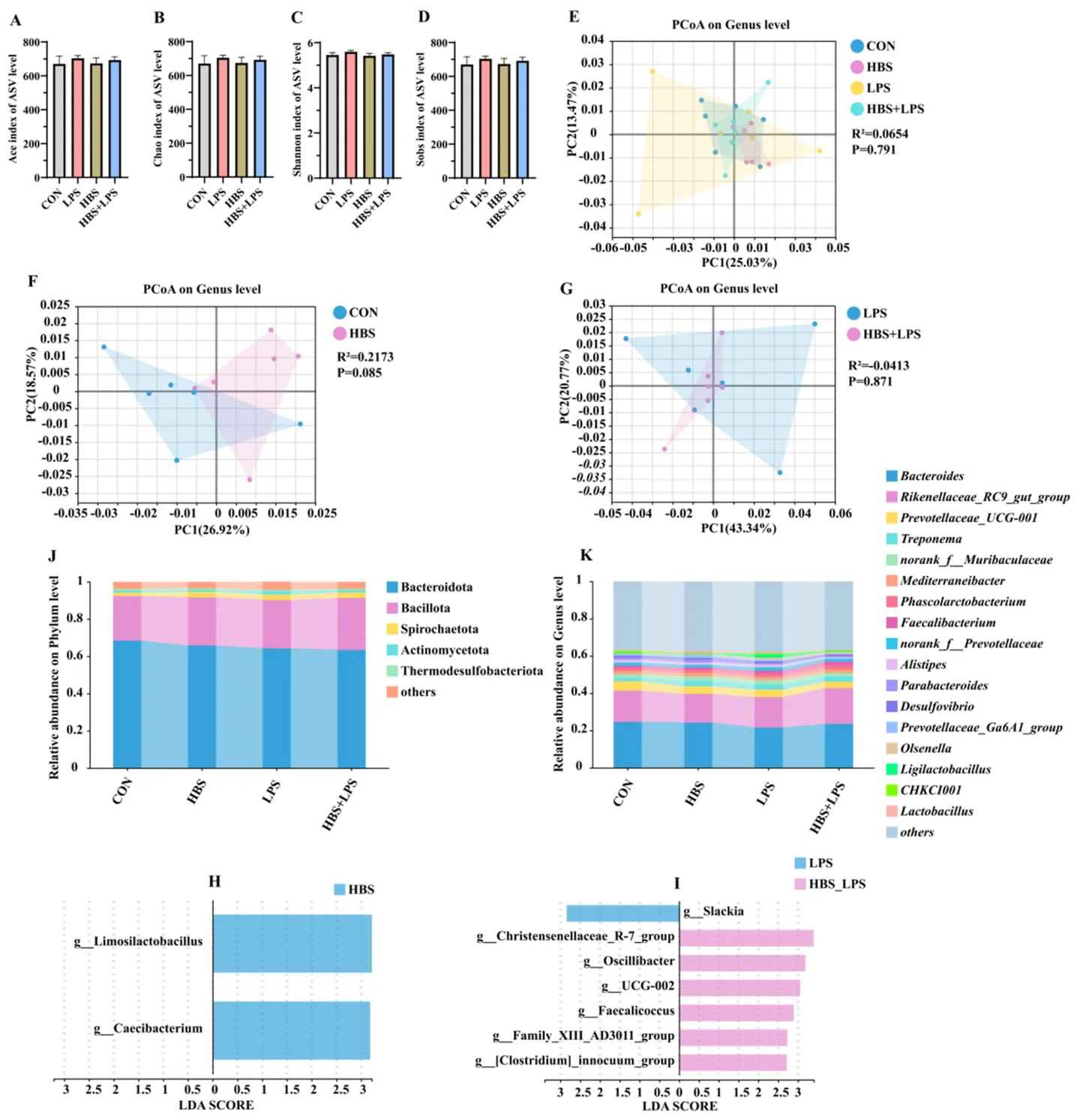
Effects of dietary HBS supplementation on the cecal microbiota of PBS- or LPS-injected laying hens: (**A**) Ace index; (**B**) Chao1 index; (**C**) Shannon index; (**D**) Sobs index; (**E**) PCoA of the four treatment groups based on Bray–Curtis distances at the genus level; (**F**) PCoA comparing CON and HBS; (**G**) PCoA comparing LPS and HBS+LPS; (**H**) treatment-associated taxa identified by exploratory LEfSe screening between CON and HBS; (**I**) treatment-associated taxa identified by exploratory LEfSe screening between LPS and HBS+LPS; (**J**) cecal bacterial composition at the phylum level; (**K**) cecal bacterial composition at the genus level. LEfSe screening used LDA > 2.5 and unadjusted *p* < 0.05; no false-discovery-rate correction was applied. CON, control; HBS, HBS supplementation; LPS, lipopolysaccharide challenge; HBS+LPS, HBS supplementation plus LPS challenge.

No significant differences in cecal α-diversity indices were detected among the groups (Figure 3A–D). PERMANOVA based on Bray–Curtis distances showed no significant differences in genus-level community structure across all groups (R^2^ = 0.0654, *p* = 0.791), between CON and HBS (R^2^ = 0.2173, *p* = 0.085), or between LPS and HBS+LPS (R^2^ = −0.0413, *p* = 0.871; Figure 3E–G). The predominant phyla and genera were similar among the groups (Figure 3J,K). Exploratory LEfSe analysis identified higher relative abundances of *Caecibacterium* and *Limosilactobacillus* in HBS than in CON, higher *Slackia* in LPS than in HBS+LPS, and higher [Clostridium]_innocuum_group, Family_XIII_AD3011_group, *Faecalicoccus*, UCG-002, *Oscillibacter*, and Christensenellaceae_R-7_group in HBS+LPS than in LPS (Figure 3H,I). Because these analyses were not corrected for multiple testing and included six birds per group, the LEfSe findings were considered exploratory.

### 2.7. Cecal Short-Chain Fatty Acid Concentrations

**Figure 4:**
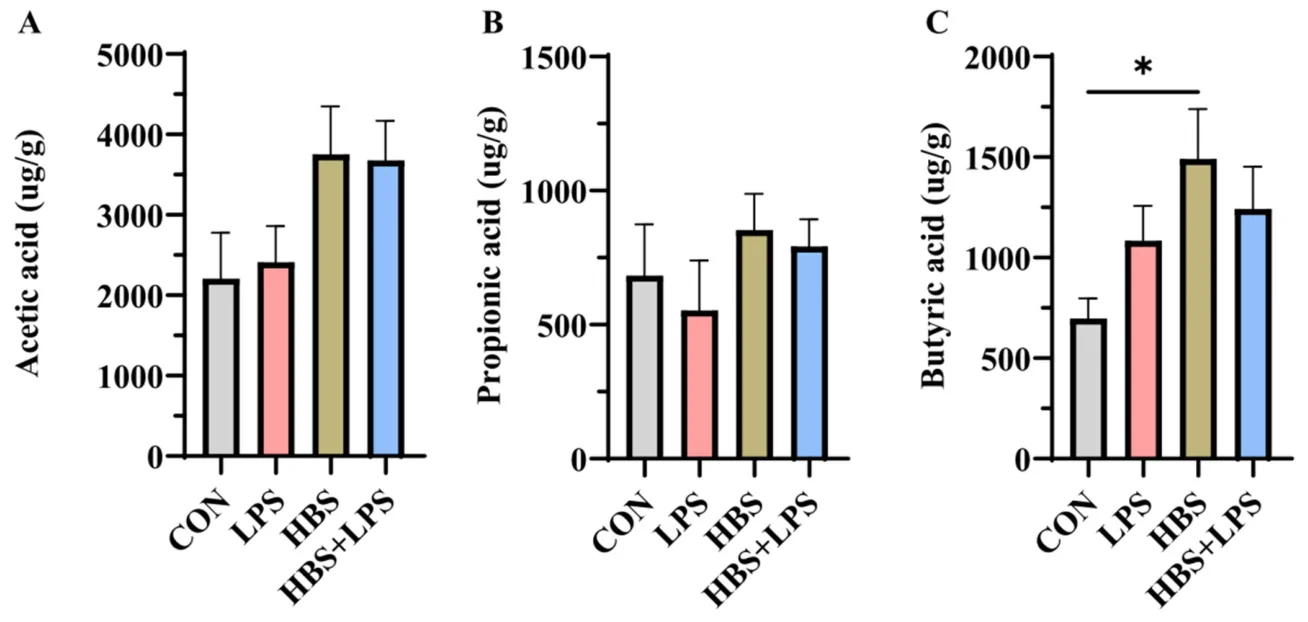
Effects of dietary supplementation with selenium-enriched *Bacillus subtilis* on cecal short-chain fatty acid concentrations in LPS-challenged laying hens: (**A**) cecal acetate concentration; (**B**) cecal propionate concentration; (**C**) cecal butyrate concentration. Data are presented as mean ± SD (*n* = 6). * *p* < 0.05. CON, control; HBS, HBS supplementation; LPS, lipopolysaccharide challenge; HBS+LPS, HBS supplementation plus LPS challenge.

Cecal acetate and propionate concentrations did not differ significantly among the four groups (Figure 4). Cecal butyrate concentration was higher in HBS than in CON (*p* < 0.05), whereas no significant difference was detected between HBS+LPS and LPS.

### 2.8. Jejunal Inflammatory, Antioxidant, and Barrier-Related Gene Expression

**Figure 5:**
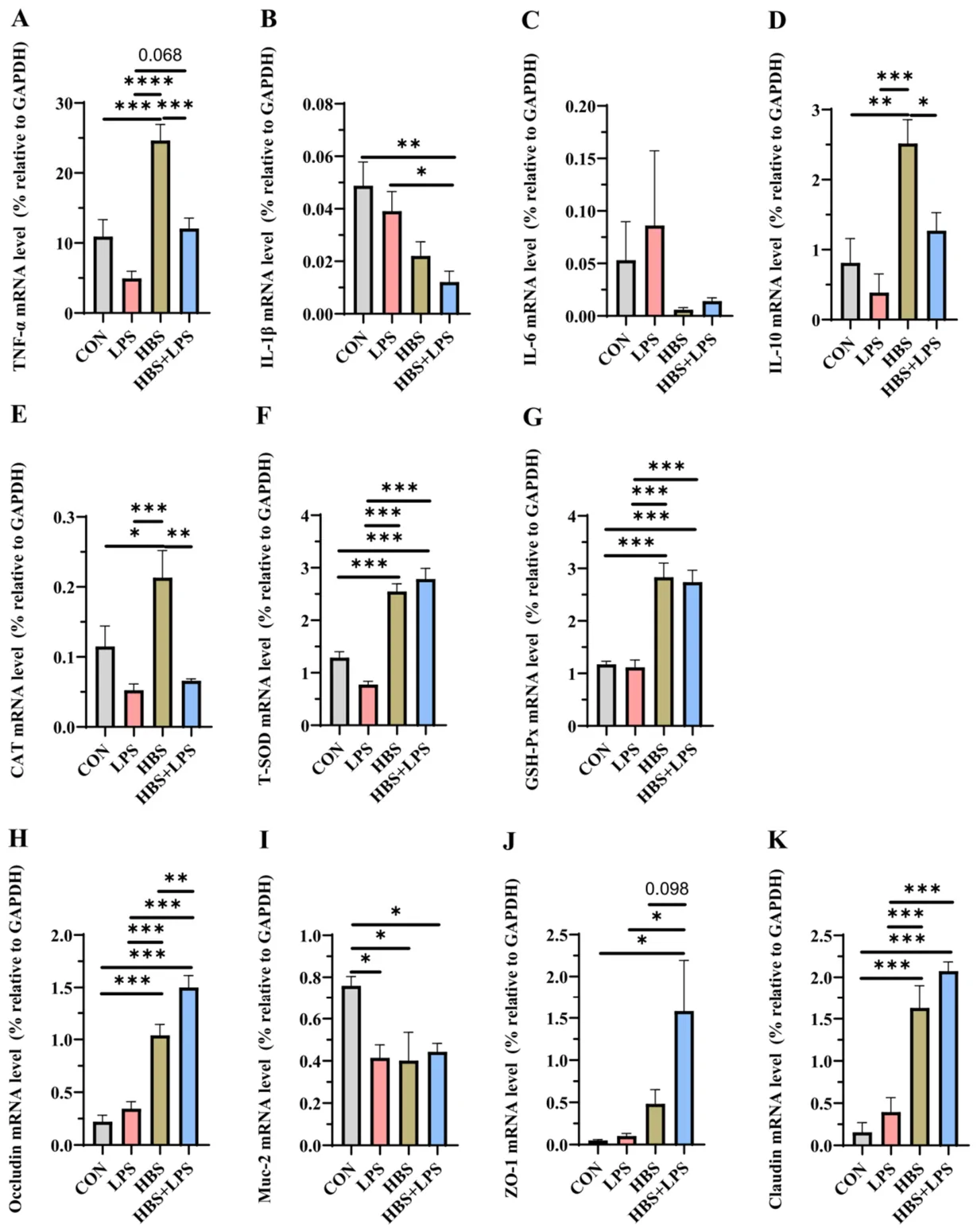
Effects of dietary supplementation with selenium-enriched *Bacillus subtilis* on jejunal inflammatory, antioxidant, and barrier-related gene expression in LPS-challenged laying hens: (**A**) TNF-α; (**B**) IL-1β; (**C**) IL-6; (**D**) IL-10; (**E**) CAT; (**F**) T-SOD; (**G**) GSH-Px; (**H**) Occludin; (**I**) MUC2; (**J**) ZO-1; (**K**) Claudin-1. Data are presented as mean ± SD (*n* = 6). * *p* < 0.05, ** *p* < 0.01, *** *p* < 0.001, and **** *p* < 0.0001. CON, control; HBS, HBS supplementation; LPS, lipopolysaccharide challenge; HBS+LPS, HBS supplementation plus LPS challenge.

As shown in Figure 5, compared with the CON group, dietary HBS supplementation significantly increased the jejunal mRNA expression of *TNF-α* (*p* < 0.001), *IL-10* (*p* < 0.01), *CAT* (*p* < 0.05), *T-SOD* (*p* < 0.001), *GSH-Px* (*p* < 0.001), *Occludin* (*p* < 0.001), and *Claudin-1* (*p* < 0.001), while decreasing *MUC2* expression (*p* < 0.05). *MUC2* expression was also lower in the LPS and HBS+LPS groups than in the CON group (*p* < 0.05). No significant difference in *IL-6* expression was observed between the CON and HBS groups. Compared with the LPS group, the HBS+LPS group exhibited lower *IL-1β* expression (*p* < 0.05) and higher *IL-10* expression (*p* < 0.05). *TNF-α* expression was numerically higher in HBS+LPS than in LPS, but the difference was not statistically significant (*p* = 0.068). In addition, the HBS+LPS group exhibited higher expression of *T-SOD*, *GSH-Px*, *Occludin*, and *Claudin-1* (*p* < 0.001) and *ZO-1* (*p* < 0.05) than the LPS group. No significant differences in *IL-6*, *CAT*, or *MUC2* expression were observed between the LPS and HBS+LPS groups. Collectively, dietary HBS supplementation enhanced the expression of several antioxidant- and tight-junction-related genes but did not uniformly suppress jejunal inflammatory gene expression.

## 3. Discussion

The present study evaluated HBS supplementation during a 12-week feeding trial and a subsequent acute LPS challenge. The principal findings were a lower feed-to-egg ratio and improved albumen quality during the feeding period, together with differences in selected serum antioxidant, inflammatory, and jejunal endpoints after LPS exposure. These findings provide evidence of responses to the HBS preparation at both the production and physiological levels. Because selenium-only and non-selenium-enriched B. subtilis groups were not included, the responses cannot be attributed to either component individually or to their interaction.

The reduction in the feed-to-egg ratio indicates a lower feed requirement per unit of egg mass. Because laying rate, feed intake, egg weight, and egg mass did not differ significantly between treatments, the production response was most clearly reflected in this integrated efficiency measure. The present findings do not identify whether altered digestion, nutrient absorption, or postabsorptive nutrient utilization contributed to the response, as these processes were not directly assessed. HBS supplementation also increased albumen height and Haugh unit, indicating improved albumen quality at the time of measurement. These changes were not accompanied by significant differences in the other measured egg-quality traits. Thus, the effects were selective rather than a general improvement across all egg-quality parameters. Oxidative stability, storage stability, albumen composition, and egg antioxidant indices were not assessed, leaving the mechanisms underlying the observed changes unresolved.

HBS+LPS showed lower liver and gizzard indices and higher serum CAT and T-SOD activities than LPS, whereas serum GSH-Px activity did not differ significantly. The enzyme responses are consistent with the involvement of selenium and probiotics in antioxidant regulation reported in previous studies [10,11,12,13,14,18,27,28,30,31,32]. The changes in relative organ weights, however, do not establish changes in organ function. Jejunal GSH-Px mRNA expression was higher in HBS+LPS than in LPS despite the absence of a corresponding difference in serum enzyme activity. This divergence may reflect tissue-specific responses, as jejunal transcript abundance and circulating enzyme activity represent different biological measurements. The inflammatory response was also cytokine-dependent. Compared with LPS, HBS+LPS showed lower serum IL-1β concentrations and jejunal IL-1β expression, together with higher jejunal IL-10 expression. In contrast, jejunal TNF-α expression was higher in HBS than in CON and was numerically higher in HBS+LPS than in LPS, although the latter difference was not significant (*p* = 0.068). Serum TNF-α did not show a corresponding significant difference. Thus, HBS modulated selected inflammatory responses without uniformly suppressing cytokine expression. Differences between local transcriptional responses and circulating cytokine concentrations, together with the single sampling time, may contribute to this pattern.

The changes in jejunal morphology were accompanied by changes in tight-junction-related gene expression. Compared with LPS, HBS+LPS showed greater villus height and a higher villus height-to-crypt depth ratio, together with higher Occludin, ZO-1, and Claudin-1 mRNA expression. Previous studies of Bacillus-based probiotics have reported effects on intestinal morphology and barrier-related responses, with outcomes dependent on strain, dose, diet, and challenge conditions [19,20,21,22,23,30,31,32]. In the present study, MUC2 expression was lower in LPS, HBS, and HBS+LPS than in CON, with no significant difference between LPS and HBS+LPS. This distinct response indicates that the transcriptional changes did not extend uniformly to all barrier-related targets. The findings support changes in jejunal architecture and tight-junction-related transcription; functional barrier integrity would require confirmation through protein-level and intestinal permeability measurements [33,34,35].

No significant differences in the measured cecal alpha-diversity indices were detected among treatments, whereas exploratory LEfSe analysis identified several treatment-associated taxa. These results suggest possible taxon-specific responses that warrant further investigation. However, relative-abundance data do not establish changes in absolute bacterial abundance or metabolic activity, and the lack of false-discovery-rate correction limits the robustness of the taxonomic findings [36,37]. Cecal butyrate concentration was higher in HBS than in CON, whereas no significant difference was detected between HBS+LPS and LPS. The difference in statistical significance between these comparisons does not establish an HBS × LPS interaction. Furthermore, the available data do not establish a microbial or butyrate-mediated explanation for the host responses observed after LPS challenge.

The present findings apply to the combined HBS preparation at a single formulation level. Without selenium-only and non-selenium-enriched B. subtilis groups, the individual contributions of selenium and the bacterium, or their potential synergy, cannot be determined. The product selenium concentration was provided by the supplier but could not be independently verified, and dietary and tissue selenium concentrations were not measured. Consequently, the actual supplemental selenium level and selenium-specific effects could not be established. Strain identity, product stability during feed preparation, and viability in the complete feed were also not determined. The challenge experiment included six sampled birds per treatment combination without an a priori power calculation, limiting the interpretation of nonsignificant findings. The retained one-way ANOVA compared treatment combinations without estimating factorial main effects or the HBS × LPS interaction and did not account for the paired subgroups within dietary replicates. These features should be considered when interpreting the challenge comparisons. Future studies should include component-specific controls, larger samples informed by power calculations, and measurements of tissue selenium concentrations and species to clarify dose-related and component-specific responses.

## 4. Materials and Methods

### 4.1. Ethics Statement

All animal procedures were approved by the Laboratory Animal Welfare and Ethics Committee of China Agricultural University, Beijing, China (approval No. AW21506202-1-01). The animal experiment was conducted at the Zhuozhou Experimental Base of China Agricultural University (Zhuozhou, China) in accordance with the approved animal welfare and ethical guidelines.

### 4.2. Experimental Design and Diets

Selenium-enriched *Bacillus subtilis* (HBS) was supplied as a lyophilized powder by Beijing Technology and Business University (Beijing, China), with a supplier-reported viable bacterial count of 1.64 × 10^6^ CFU/g. The HBS-supplemented diet was formulated to provide 0.4 mg supplemental Se/kg diet. Lipopolysaccharide (LPS; *Escherichia coli* O55:B5) was purchased from Sigma-Aldrich (St. Louis, MO, USA). The ingredient composition and nutrient levels of the basal diet are presented in Table S1.

A total of 120 healthy 54-week-old Jinghong-1 laying hens with similar body weights were obtained from the Zhuozhou Experimental Base of China Agricultural University (Zhuozhou, China). Birds were randomly assigned to one of two dietary treatments: a basal diet (CON) or the basal diet supplemented with HBS based on the formulation target described above. Each treatment consisted of six biological replicates, with 10 hens per replicate housed in five cages (two hens per cage). Throughout the 12-week feeding trial, hens were maintained in an environmentally controlled facility at 25 ± 2 °C and 50 ± 10% relative humidity under a 16 h light/8 h dark photoperiod, with ad libitum access to feed and water.

On the final day of the 12-week feeding period, hens were fasted for 12 h. Within each original dietary replicate, the 10 hens were allocated to two challenge subgroups of five hens. One subgroup received an intraperitoneal injection of phosphate-buffered saline (PBS), whereas the other received LPS at 0.5 mg/kg body weight. This produced four treatment combinations: CON (basal diet + PBS), LPS (basal diet + LPS), HBS (HBS-supplemented diet + PBS), and HBS+LPS (HBS-supplemented diet + LPS), each comprising six replicate subgroups of five hens (30 injected birds per treatment combination). Twelve hours after injection, one hen was randomly selected from each replicate subgroup (*n* = 6 sampled birds per treatment combination), weighed, and humanely euthanized. Blood, liver, jejunum, and cecal contents were collected immediately. Serum was separated by centrifugation and stored at −80 °C. Tissue samples intended for histological examination were fixed in 4% paraformaldehyde, whereas samples for gene expression and cecal content analyses were stored at −80 °C until analysis.

The experimental unit varied by endpoint. For laying performance, each replicate of 10 hens housed in five cages was the experimental unit (*n* = 6 per dietary treatment). For egg-quality traits, measurements from five eggs within each replicate were averaged to obtain one replicate-level value. For the challenge experiment, each dietary replicate was divided into PBS- and LPS-treated subgroups of five hens. One bird was sampled from each subgroup for serum, organ, histological, gene expression, microbiota, and SCFA analyses, resulting in six observations per dietary × challenge combination. The PBS- and LPS-treated subgroups originated from the same dietary replicates, and each sampled bird retained its original dietary-replicate membership. The sample size was based on the six original dietary replicates; no a priori power calculation was performed.

### 4.3. Laying Performance and Egg Quality

Laying performance was evaluated throughout the 12-week feeding trial. Egg production and total egg weight were recorded daily for each replicate, whereas feed consumption was recorded weekly on a replicate basis. Laying rate, average egg weight, egg mass, and average daily feed intake (ADFI) were calculated using standard poultry production equations. The feed-to-egg ratio was calculated for each replicate as total feed intake divided by total egg mass over the corresponding period; a lower value indicates more efficient feed utilization.

At the end of the feeding trial, five eggs were randomly collected from each replicate (30 eggs per treatment) for egg-quality evaluation. Egg weight, eggshell strength, albumen height, Haugh unit, yolk color, and eggshell thickness were measured using an Egg Analyzer™ and an Egg Force Reader (Orka Food Technology Ltd., Ramat HaSharon, Israel) according to the manufacturer’s instructions. The shell, yolk, and albumen were separated, and their respective weights were determined using an electronic analytical balance. The five egg measurements within each replicate were averaged before statistical analysis so that the dietary replicate, rather than the individual egg, remained the experimental unit.

### 4.4. Organ Index Determination

At 12 h after PBS or LPS injection, the selected laying hens were weighed individually and then humanely euthanized by cervical dislocation. The liver, spleen, gizzard, proventriculus, and kidneys were immediately excised, carefully cleared of adhering connective tissue and contents, rinsed with ice-cold sterile physiological saline, and blotted dry with filter paper. Each organ was weighed using an electronic analytical balance. The relative organ index was calculated as the percentage of organ fresh weight relative to live body weight using the following equation: Organ index = (organ fresh weight/live body weight) × 100%.

### 4.5. Intestinal Histomorphometry

Approximately 0.5 cm segments of the jejunum and cecum were collected immediately after euthanasia and fixed in 4% paraformaldehyde for 24 h. The tissues were dehydrated through a graded ethanol series, cleared in xylene, embedded in paraffin, sectioned at a thickness of 5 μm using a rotary microtome, and stained with hematoxylin and eosin (H&E) according to the manufacturer’s instructions (Wuhan Servicebio Technology Co., Ltd., Wuhan, China). Histological sections were digitized using a whole-slide scanner and analyzed with CaseViewer 2.4 software (3DHISTECH Ltd., Budapest, Hungary). Representative images of the jejunum and cecum were captured under identical imaging conditions. For jejunal morphometric analysis, well-oriented villus–crypt units were randomly selected from each section. Villus height was measured from the tip of the villus to the villus–crypt junction, whereas crypt depth was measured from the villus–crypt junction to the base of the crypt using ImageJ software (National Institutes of Health, Bethesda, MD, USA; https://imagej.nih.gov/ij/, accessed on 30 September 2026). The villus height-to-crypt depth ratio (VH/CD) was subsequently calculated. The average value obtained for each bird was used for statistical analysis. Representative cecal sections were qualitatively examined to assess mucosal morphology, epithelial integrity, and inflammatory changes.

### 4.6. Serum Antioxidant and Cytokine Analyses

Blood samples were collected from the wing vein and allowed to clot at room temperature for 30 min. The samples were then centrifuged at 3000× *g* for 15 min at 4 °C, and the resulting serum was collected and stored at −80 °C until analysis.

Serum concentrations of the pro-inflammatory cytokines tumor necrosis factor-α (TNF-α) and interleukin-1β (IL-1β) were determined using commercially available enzyme-linked immunosorbent assay (ELISA) kits (Nanjing Jiancheng Bioengineering Institute, Nanjing, China) according to the manufacturer’s instructions. The absorbance of each sample was measured using a microplate reader (BioTek Instruments, Winooski, VT, USA), and cytokine concentrations were calculated from standard calibration curves. Serum antioxidant status was evaluated by measuring the activities of catalase (CAT), glutathione peroxidase (GSH-Px), and total superoxide dismutase (T-SOD) using commercial assay kits (Nanjing Jiancheng Bioengineering Institute, Nanjing, China) according to the manufacturer’s protocols. Absorbance was determined using a microplate reader (BioTek Instruments, Winooski, VT, USA), and enzyme activities were calculated according to the manufacturer’s instructions.

### 4.7. Quantitative Real-Time PCR

Total RNA was extracted from jejunal tissue using the FastPure Cell/Tissue Total RNA Isolation Kit V2 (Vazyme Biotech Co., Ltd., Nanjing, China) according to the manufacturer’s instructions. The concentration and purity of the extracted RNA were determined using a NanoDrop 2000 spectrophotometer (Thermo Fisher Scientific, Wilmington, DE, USA). RNA samples of acceptable concentration and purity were used for subsequent reverse transcription. Complementary DNA (cDNA) was synthesized from total RNA using HiScript IV RT SuperMix for qPCR (+gDNA Wiper) (Vazyme Biotech Co., Ltd.). Quantitative real-time PCR (qRT-PCR) was performed using Taq Pro Universal SYBR qPCR Master Mix (Vazyme Biotech Co., Ltd.) on a CFX96 Real-Time PCR Detection System (Bio-Rad Laboratories, Hercules, CA, USA). The amplification program consisted of an initial denaturation at 95 °C for 30 s, followed by 40 cycles of denaturation at 95 °C for 10 s and annealing/extension at 60 °C for 30 s. Melting curve analysis was performed after amplification to verify the specificity of the PCR products. In jejunal tissues, the mRNA levels of inflammation-related genes (TNF-α, IL-1β, IL-6, and IL-10), antioxidant-related genes (CAT, T-SOD, and GSH-Px), and intestinal barrier-related genes (Occludin, MUC2, ZO-1, and Claudin-1) were determined. GAPDH was used as the endogenous reference gene. The expression level of each target gene was normalized to that of GAPDH and expressed as a percentage relative to GAPDH. The primer sequences used for qRT-PCR are provided in Table S2.

### 4.8. Cecal Microbiome Analysis

Cecal contents were aseptically collected immediately after euthanasia, snap-frozen in liquid nitrogen, and stored at −80 °C until DNA extraction. Total microbial genomic DNA was extracted using the Mag-Bind^®^ Stool DNA Kit (Omega Bio-Tek, Norcross, GA, USA) according to the manufacturer’s instructions. DNA concentration and purity were determined using a NanoDrop 2000 spectrophotometer (Thermo Fisher Scientific, Wilmington, DE, USA), and DNA integrity was verified by 1% agarose gel electrophoresis.

The V3–V4 hypervariable regions of the bacterial 16S rRNA gene were amplified using the universal primer pair 338F (5′-ACTCCTACGGGAGGCAGCAG-3′) and 806R (5′-GGACTACHVGGGTWTCTAAT-3′) with Phusion High-Fidelity PCR Master Mix (New England Biolabs, Ipswich, MA, USA). PCR amplification was performed under the following conditions: initial denaturation at 95 °C for 3 min, followed by 27 cycles of denaturation at 95 °C for 30 s, annealing at 55 °C for 30 s, and extension at 72 °C for 45 s, with a final extension at 72 °C for 10 min. PCR products were purified using a MinElute^®^ PCR Purification Kit (QIAGEN, Hilden, Germany), quantified, pooled in equimolar concentrations, and subjected to paired-end sequencing (2 × 300 bp) on an Illumina MiSeq PE300 platform (Illumina, San Diego, CA, USA) by Majorbio Bio-Pharm Technology Co., Ltd. (Shanghai, China).

### 4.9. Short-Chain Fatty Acid Determination by Gas Chromatography

The concentrations of short-chain fatty acids (SCFAs), including acetate, propionate, and butyrate, in cecal contents were determined by gas chromatography (GC) using a Shimadzu GC-2010 system (Shimadzu Corporation, Kyoto, Japan). Briefly, approximately 150 mg of cecal contents was homogenized with 1.5 mL of ultrapure water and centrifuged at 5000× *g* for 10 min at 4 °C. Subsequently, 500 μL of the supernatant was mixed with 100 μL of 25% metaphosphoric acid, incubated at 4 °C for 30 min, and centrifuged again at 5000× *g* for 10 min at 4 °C. Following centrifugation, 500 μL of the resulting supernatant was transferred into a clean tube and mixed with 490 μL of ultrapure water and 10 μL of crotonic acid as an internal standard. After thorough vortex mixing, the solution was filtered through a 0.22 μm membrane filter and transferred into a 1.5 mL glass vial for GC analysis. Individual SCFAs were identified by comparing their retention times with those of authentic standards and quantified using calibration curves based on analyte-to-internal-standard peak-area ratios.

### 4.10. Bioinformatics Analysis

Raw sequencing reads were demultiplexed, quality-filtered using fastp (v0.19.6), and merged using FLASH (v1.2.11). High-quality sequences were denoised using the DADA2 plugin implemented in QIIME2 (version 2024.10) to generate amplicon sequence variants (ASVs). Taxonomy was assigned to representative ASV sequences using the SILVA 16S rRNA database (version 138.2) with a naïve Bayes classifier. Alpha-diversity indices, including Ace, Chao1, Shannon, Sobs, and Good’s coverage, were calculated using Mothur (version 1.30.2). Bray–Curtis dissimilarities were calculated at the genus level and visualized using principal coordinate analysis (PCoA), and PERMANOVA was used to evaluate overall and prespecified pairwise differences in community structure. Differential taxa were screened using LEfSe with LDA > 2.5 and unadjusted *p* < 0.05. No false-discovery-rate correction was applied; the identified taxa were therefore interpreted as exploratory associations.

### 4.11. Statistical Analysis

Data are presented as means ± standard deviations unless otherwise indicated in the table or figure legends. Replicate-level values from the two-group feeding experiment were compared using a two-tailed unpaired Student’s *t*-test. Data from the four-group LPS-challenge experiment were analyzed by one-way analysis of variance followed by Duncan’s multiple range test when the overall test was significant. No multiplicity adjustment was applied across gene expression endpoints; these analyses were considered exploratory. Figures were prepared using GraphPad Prism 9.5. *p* < 0.05 was considered statistically significant. The symbols *, **, ***, and **** indicate *p* < 0.05, *p* < 0.01, *p* < 0.001, and *p* < 0.0001, respectively.

## 5. Conclusions

Dietary HBS supplementation reduced the feed-to-egg ratio and improved albumen height and Haugh unit in laying hens. Following LPS challenge, HBS supplementation was associated with lower serum IL-1β concentrations, higher CAT and T-SOD activities, improved jejunal villus morphology, and increased expression of selected antioxidant- and tight-junction-related genes. However, inflammatory gene responses were not uniformly suppressed, and no significant differences were detected in overall cecal microbial diversity or community structure. Cecal butyrate concentration was increased by HBS under non-challenged conditions. Overall, these findings indicate that HBS may improve feed utilization and modulate selected antioxidant, inflammatory, and intestinal responses in laying hens. Because selenium-only and non-selenium-enriched B. subtilis controls were not included and selenium exposure was not independently verified, the component-specific effects of HBS require further investigation.

## Figures and Tables

**Table 1:** Effects of dietary supplementation with selenium-enriched *Bacillus subtilis* on the laying performance of laying hens.

Group	Egg-Laying Rate (%)	Egg Weight (g)	Egg Mass (g)	Average Daily Feed Intake (g)	Feed-to-Egg Ratio
CON	85.24	65.64	55.91	119.51	2.15 ^b^
HBS	91.94	64.12	58.93	115.38	1.96 ^a^
*p*-value	0.095	0.093	0.182	0.073	0.023

Values are treatment means based on six independent replicate groups per treatment; each replicate group contained 10 hens housed in five cages. Feed-to-egg ratio was calculated as total feed intake divided by total egg mass at the replicate level. Within a column, means with different superscript letters differ significantly (*p* < 0.05). ADFI, average daily feed intake; CON, basal diet; HBS, HBS-supplemented diet.

**Table 2:** Effects of dietary supplementation with selenium-enriched *Bacillus subtilis* on the egg quality of laying hens.

Group	Egg Weight (g)	Eggshell Strength (N)	Albumen Height (mm)	Yolk Color	Haugh Unit	Eggshell Weight (g)	Yolk Weight (g)	Albumen Weight (g)	Eggshell Thickness (mm)
CON	63.40 ± 1.18	37.29 ± 1.39	6.60 ± 0.18 ^a^	8.76 ± 0.23	78.65 ± 1.38 ^a^	7.98 ± 0.11	16.85 ± 0.51	38.57 ± 1.17	0.37 ± 0.01
HBS	64.65 ± 1.50	37.76 ± 1.72	7.17 ± 0.33 ^b^	8.52 ± 0.27	83.01 ± 1.78 ^b^	8.10 ± 0.14	17.06 ± 0.33	39.48 ± 1.31	0.36 ± 0.01
*p*-value	0.372	0.407	0.003	0.163	0.001	0.105	0.477	0.572	0.787

Values are replicate-level means ± SD (*n* = 6 dietary replicates per treatment). Five eggs measured within each replicate were treated as subsamples and averaged before analysis. Within a column, means with different superscript letters differ significantly (*p* < 0.05). CON, basal diet; HBS, HBS-supplemented diet.

**Table 3:** Effects of dietary supplementation with selenium-enriched *Bacillus subtilis* on organ indices of LPS-challenged laying hens.

Group	Liver (%)	Spleen (%)	Gizzard (%)	Proventriculus (%)	Kidney (%)
CON	2.29 ± 0.15 ^ab^	0.13 ± 0.02	1.91 ± 0.22 ^b^	0.38 ± 0.05	0.38 ± 0.07
HBS	2.06 ± 0.30 ^a^	0.14 ± 0.02	1.67 ± 0.18 ^a^	0.37 ± 0.06	0.40 ± 0.06
LPS	2.65 ± 0.50 ^b^	0.17 ± 0.02	2.27 ± 0.16 ^c^	0.33 ± 0.04	0.36 ± 0.03
HBS+LPS	2.23 ± 0.17 ^a^	0.14 ± 0.03	1.83 ± 0.07 ^ab^	0.38 ± 0.03	0.39 ± 0.07
*p*-value	0.025	0.081	<0.001	0.246	0.603

Values are presented as mean ± SD (*n* = 6). Within a column, means with different superscript letters differ significantly (*p* < 0.05). CON, control; HBS, HBS supplementation; LPS, lipopolysaccharide challenge; HBS+LPS, HBS supplementation plus LPS challenge.

## Data Availability

The data supporting the findings of this study are available within the article and its Supplementary Materials. The raw 16S rRNA sequencing data have been deposited in a public repository.

## Supplementary Materials

The following supporting information can be downloaded at https://www.mdpi.com/article/10.3390/ijms27198778/s1.
