# Supplementary material for: Dietary Selenium-Enriched Bacillus subtilis Improves Feed Utilization and Modulates Selected Responses to LPS Challenge in Laying Hens
Source: Int J Mol Sci. 2026 Sep 30;27(19):8778. doi: 10.3390/ijms27198778 (PMC13665395; doi:10.3390/ijms27198778)
Supplement: Supplementary file 1 [file ijms-27-08778-s001.zip › ijms-4513204-supplementary.pdf]

Supplementary Tables

Table S1. Composition and nutrient levels of the basal diet (% as-fed basis, unless otherwise indicated).

**Ingredient**	**Amount (%)**
Corn	65.05
Soybean meal	24.00
Limestone	8.20
Dicalcium phosphate	1.70
Salt	0.30
DL-Methionine	0.12
Trace mineral premix	0.30
Multivitamin premix	0.03
Choline chloride	0.10
Total	100
Nutrient levels	
Crude protein	16.04
Metabolizable energy (Mcal/kg)	2.69
Calcium	3.60
Total phosphorus	0.65
Non-phytate phosphorus	0.39
Methionine	0.38
Methionine and cystine	0.65
Lysine	0.78
Tryptophan	0.16
Threonine	0.59

The trace mineral premix supplied 6.8 mg/kg copper, 66 mg/kg iron, 83 mg/kg zinc, 80 mg/kg manganese, and 1 mg/kg iodine.
The vitamin premix supplied 11,700 IU/kg vitamin A, 3600 IU/kg vitamin D_3, 21 IU/kg vitamin E, 4.2 mg/kg vitamin K_3, 3 mg/kg thiamine, 10.2 mg/kg riboflavin, 0.9 mg/kg folic acid, 15 mg/kg calcium pantothenate, 45 mg/kg nicotinic acid, 5.4 mg/kg pyridoxine, 24 μg/kg vitamin B_{12}, and 150 μg/kg biotin.

Table S2. Primer sequences used for RT-qPCR.

Gene	Primer sequence (5′–3′)
cTNF-α	F: CGTCAGTGTCCCACAGACAT<br>R: GAGCAGGCAGGCTCCTAAAA
cIL-1β	F: ACTGGGCATCAAGGGCTA<br>R: GGTAGAAGATGAAGCGGGTC
cIL-6	F: CGCCCAGAAATCCCTCCTC<br>R: AGGCACTGAAACTCCTGGTC
cIL-10	F: GCTGCCAAGCCCTGTT<br>R: CCTCAAACTTCACCCTCA
cZO-1	F: CTTCAGGTGTTTCTCTTCCTCCTC<br>R: CTGTGGTTTCATGGCTGGATC
cMUC-2	F: ATGCGATGTTAACACAGGACTC<br>R: GTGGAGCACAGCAGACTTTG
cOccludin	F: ACGGCAGCACCTACCTCAA<br>R: GGGCGAAGAAGCAGATGAG
cClaudin	F: CATACTCCTGGGTCTGGTTGGT<br>R: GACAGCCATCCGCATCTTCT
cCAT	F: GGCAACCCAATAGGAGATAAGC<br>R: TCCTTTCGCATGCACAACTC
cT-SOD	F: TGTGCATGAATTTGGAGACAAC<br>R: TTGCAGTCACATTGCCGAG
cGSH-PX	F: GACCAACCCGCAGTACATCA<br>R: GAGGTGCGGGCTTTCCTTTA
cGAPDH	F: GTCCAAGTGGTGGCCATCAA<br>R: GCTGAGGGAGCTGAGATGAT
